# 

**Published:** 1887-10-01

**Authors:** 


### The Hospital

A Weekly Institutional Journal of

### Science, Medicine, Nursing, and Philanthropy.

EDITED BY

HENRY C. BURDETT,

AUTHOR OF "HOSPITALS AND THE STATE"; "PAY HOSPITALS OF THE WORLD"; "COTTAGE HOSPITALS, GENERAL, FEVER, AND CONVALESCENT, WITH FIFTY BEDS AND UNDER: THEIR ORGANISATION, MANAGEMENT, AND WORK"; ETC., ETC.

ACTING EDITOR:

GEORGE W. POTTER, M.D.,

AUTHOR OF PAPERS ON "BACTERIOLOGY"; "SCARLET-FEVER"; "RHEUMATISM"; ETC., ETC.

SURGEON GEN'L'S OFFICE LIBRARY

VOL. III. FOR 1887-88.

(1st October, 1887, to 31st March, 1888.)

LONDON:

PUBLISHED BY "THE HOSPITAL" (Limited) 38, OLD JEWRY, E.C.

W1
HO69X

### INDEX TO VOL. III. 1887-8.

Aberdeen, Combination Hospital for, 319
—— Royal Infirmary, 220
Accidents, the Common, of Everyday Life: Accident to a Child in a Perambulator, 210
—— Bones, Fractures, &c., 275
—— Burns and Scalds, 152, 238
—— Fractures and Dislocations, 307, 389
—— Greenstick Fracture, 275
—— Infancy, 12, 76, 110, 275
Aids to Nursing, 313, 331, 347, 363, 379, 395, 409, 425, 441
Alcohol, Statistics of Consumption in Hospitals, 17
Almanac, THE HOSPITAL, for 1888, 220, 250
American Lunatic Asylums, 365
Amusements with Profit, 18, 50, 106, 140, 157, 212, 242, 260, 310, 326, 342, 358, 374, 390, 404, 436
Animal Life, Incidents of, 101, 240
Apprentices' Exhibition at People's Palace, 202
Architects, Hospitals, and Asylums, 102
Are We a Capable People? 169
Asylums, Round About the, 365, 416, 424

Bacteriology, 3
Bath Eye Infirmary and the Guardians of the Poor, 334
Battle of the "Pathies," the, 278
Bed-room Fires, the Science of, 361
Bees and the Hive, 423
Belfast, proposed Consumption Hospital, 413
Biology of the Future, the, 274
Birching Machine, a, 321
Birmingham, Queen's Hospital, 26
Birmingham Provident Dispensaries, 412
Blindness and Artificial Light, 320
Boarding Out of Pauper Children, 221
Board School Children and the Liverpool Dispensaries, 383
Boiler Makers, and the Queen's Jubilee, 235
Bolton Infirmary, Bequest to, 335
Bone Setting in the Black Country, 431
Bournemouth, Victoria Jubilee Hospital, 383
Brains at a Premium, 345
Bread, Whole Meal, 223
Bread and Food Reform League, 223
Breakfast, Hints About, 341
Bricks or Daisies, 385
Bridlington Medical Relief Club, 149
Bristol, Extracts from Records of St. Peter's Hospital, 114
Bristowe, Dr. J. S., and the Hospitals Association, 268
*British Medical Journal* and the Hospitals Association, 80
British Nurses' Association, 202, 351, 397, 405, 406, 419, 434, 438
Brompton Hospital for Consumption, 181
Brompton Cancer Hospital, 398
Buchanan, Miss, the Case of, 259
Buffalo State Asylum, 365
Burdett, Mr. H. C., and the National Pension Fund, 287
Butter or Butterine, 45
Buxton Bath Charity, 284

Calculi, Norwich Collection of, 324, 380
Can a Man of Science Wonder? 415
Cancer Hospital, Brompton, 398
Charitable Bequests during 1887, 412
Charitable Funds, Cost of Collecting, 9
Charitable, the Morals of the, 353
Charity Dispensing Society, 287
Charity Organization Society, 27
Chelmsford, Infectious Diseases Hospital at, 220
Chelsea Hospital for Women, 334
Chest, City of London Hospital for Diseases of the, 398
Children, Society for the Prevention of Cruelty to, 222
Children's Dresses, 237
Children's Paradise, a, 226
Christmas in the Hospitals, 203, 241, 254, 265, 280, 304
Christmas Pudding, the, 216
Churches, Chapels, and People in London, 189
Churchill, Lord R., and St. Mary's Hospital, 412
Circular Wards, 61, 98
City of London Lying-In Hospital, 350
City of London Hospital for Diseases of the Chest, 398
Clayton Hospital, Wakefield, 9
Clinical and Therapeutic Notes, 95, 165, 199, 233
Climate, a Dangerous, 117
Cock Fighting, 369
Colchester Hospital, 202, 203
Cold Feet, 55
Collecting Boxes in Schools, 367
Colour Blindness in the Mercantile Marine, 135
Combination Hospital for Aberdeen, 319
Compliments of the Season, 203
Comparative Expenditure of Hospitals, 232
Complaints Against Hospitals, 236
Conscience, the Market Value of, 343
Consumption, Climatic Treatment of, [illegible]4
Consumption, a Specific for, 182
Consumption Hospital, Brompton, 181
Consumption Hospital for Belfast, 413
Consumption, National Hospital for, 234, 412
Convalescents, Amusements for, 58, 124, 211, 230, 322, 386, 440
Convalescent Home for Metropolitan Police, 428
Convalescent Homes in the Eastern Counties, 338 346
Co-operation, Progress in, 237
Cook, a word with the, 298
Cooks, Lady, for Hospitals, 12, 87, 143, 178
Coroners, Legal or Medical, 29
Cottage Hospitals, 152, 321
County Hospital, a, 209
Cows for Cottagers, 401
Crockery, Gift of, to Liverpool Hospitals, 235
Crown Prince of Germany, Illness of the, 125, 391
Cumberland and Westmoreland Asylum, 424

Dairies, Old and New, 164
Dead by the Way, 344
Dental Hospital of London, 444
Dentists' Chair, Dangers of the, 81
Deep Sea Fishermen, Mission to, 253, 269
Derbyshire General Infirmary, and Bishop of Liverpool, 132
Devonshire Hospital, and Rheumatism, 284
Dishonour, an Indelible, 169
Doctor, a, and his Hobby, 223
Doctors, State Supported, 183
Doctors' Pulpit, the:
—— A Little Knowledge, 266
—— Bacteriology, 3
—— Supper and Sleep, 21
—— Cold Feet, 55
—— Scarlet Fever, 75
—— Woman at Home, 91, 109 127
—— Doctors and Patients, 144, 161
—— The Physic Bottle, 175
—— The Spirit Bottle, 197
—— The Christmas Pudding, 216
—— Men and Marriage, 231
—— Every-day Science, 248
—— A Word with the Cook, 298
—— Nursery Cares, 315
—— The Medicine of Work, 329
—— Brains at a Premium, 345
Doctor's Pulpit, the:
—— The Science of Bed-room Fires, 361
—— An Apology for the East Wind, 377
—— Penalties of Weakness, 393
—— The Morals of the Workshop, 407
—— The Bees and the Hive, 423
—— The Backbones of the Poor, 439
Doctors, Brief Life for, 63
Doctors and Patients, 144, 161
Doctors, Lady, in France, 81
Doctors in Cotton Wadding, 385
Dreams, what they are Made of, 353
Drugs and Remedies, Present, 16, 131
Drug Laboratories, Modern, 129, 162
Dublin, City of, Hospital, 133
Dublin, National Children's Hospital, 220
Ducker's Portable Hospitals, 334

Early Marriages, 369
East Riding Asylum, 416
East Wind, an Apology for, 377
East and West, by Leslie Keith, 13, 31, 47, 68, 85, 104, 119, 137, 153, 171, 185, 207, 224
East London Nursing Society, 383, 396
Eastern Counties, Convalescent Homes in, 338, 346
Edinburgh Children's Hospital, 318
—— Medical Missionary Society, 367
—— New Lunatic Asylum for, 382
—— Saltwater Supply for, 151
Egg-phosphate for Overstrained Nerves, 302
Electricity, a Remedy, 16
Emigration and Self-Help, 431
Ennis District Asylum, 424
Epilepsy and Responsibility, 253
Everybody's Page, 270, 286, 320, 336, 352, 386, 384, 400, 415, 430
Every-day Science, 248
Examinations for Nurses, are they Desirable, 303, 325
Executors and Hospital Letters, 269
Exeter Borough Asylum, 424
Expenditure, Hospital, Comparative Statement of, 232

Fever, why Die of? 63
Fever Hospital, a New, 135
Fingers and Brains, 141
Fish as Brain Food, 302
Floating Hospital in the North Sea, 269
Food, the Digestibility of, 45
Foods, Liquid, 84
French Hospital, the, 284
From Friends across the Sea, 93, 145

Gateshead Children's Hospital, 220
Genius, the Origin of, 271
Gentleman with a Grievance, a, 107
German Crown Prince, Illness of, 125, 391
Ghastly Experience, a, 239
Gladstone, Mr., and Scarlet Fever Convalescents 215, 220
Glasgow, Proposed New Hospital for, 26
—— Royal Infirmary, 335
—— Royal Asylum, 416
—— Hospital for Sick Children, 378
*Globe*, the, and Hospital Patients, 236
Go Thou and Do Likewise, 99

Granny, 63
Great Northern Central Hospital, 44, 99, 116, 300, 398, 413
Habits, the Evil Effects of Regular, 97
Harrisburg, Penn. State Lunatic Asylum, 365
Hastings, New Hospital at, 42, 61, 168
Healing the Sick, 37
Health and Precocity, 135
Health Lectures for the People, 201
Heredity, Influence of, 223
Hindu Medicine, 321
Hints About Breakfast, 341
"Holiday House" at Edinburgh, 335
Holmes, Oliver Wendell, 5, 23, 57
Home Colonization, 385
Home Rule for Nurses, 227
Hospital Construction, 121, 139, 156, 188
Hospital Depression, and the Registration of Nurses, 56
Hospital Finances, cause of Depressed Condition of, 9
Hospital Life, Recollections of, 177, 241
Hospital Patients, a Nurse's View of, 49
Hospital Physician, £200 a Year for, 203
"Hospital," Almanac, the, 250
Hospital Management and Methods of Raising Incomes, 257, 264, 288, 296, 314
HOSPITAL, THE, and the *Lancet*, 183, 287
——— and the *Echo*, 98
——— ——— Permanent Enlargement of, 444
Hospital Gates, at the, 299
Hospital Saturday Fund, Metropolitan, 284, 366
——— Bolton, 301
——— Coventry, 148
——— Eastbourne, 8
——— Exeter, 251
——— Heckmondwike, 251
——— Leeds, 300
——— Liverpool, 132
——— Manchester, 182
——— Salisbury, 26
——— Wolverhampton, 200
Hospital Statistics, 33, 232
Hospital Sunday Fund, Metropolitan, 116, 134, 200, 300
——— Birmingham, 115, 300
——— Hull, 116, 250
——— Keighley, 236
——— Liverpool, 132, 285
——— Londonderry, 150
——— Manchester, 182
——— Newcastle-on-Tyne, 8, 115
——— Richmond, 202
——— Sheffield, 351
——— Southport, 382
——— For India, 444
Hospitals Association, 26, 43, 59, 78, 116, 149, 202, 268, 284, 303, 382, 398
——— Meeting of the Council, 284
——— Third Annual Report, 96
——— and *British Medical Journal*, 80
——— and THE HOSPITAL, 180
Hospitals and Provident Dispensaries, Union proposed between, 200, 401
Hospitals and the *Pall Mall Gazette*, 107, 134
Hospitals and the Public, 73
Hospitals, New Sources of Income for, 191
Hospitals, Our, as Patients Find Them, 22
Hospitals' Week, 200
Hull Infirmary, 62
——— Working-men Governors, 150
Hydrobromates, The, 190

Incompleteness, 427
Incurables, Hospital for, 325
India, Nursing Employment in, 389
India, National Association for Supplying Female Medical Aid to the Women of, 445
Indian Women, Women Doctors for, 71, 143
Infectious Diseases, Compulsory Notification of, 11
Infectious Diseases Hospitals, 236
Injured, First Aid to the, 9
Inquests, Farcical, 99
Insane, Reminiscences of the, 187
Insane, a Plea for the, 302
Institute of Medical Electricity, 382
Intelligent Reciprocity, 99
Intelligent Woman of the Period, the, 337
Irish Distressed Ladies, 447

Jeyes' Disinfectants, 303
Jewish Hospital and Orphan Asylum, 428
Jubilee, Schemes for Celebrating the Queen's, 235, 318

King's College Hospital, 318
Kingsford, Death of Dr. Anna, 383, 419
Kitchen Work and Probationers, 62
Knowledge, A Little, 266

Ladies Physic, and Mr. Ruskin, 29
Lady Doctors in France, 81
*Lancet*, The, Reported Change in Proprietorship, 399
——— and THE HOSPITAL, 183, 287
——— and the College of Physicians, 431
Layman, a, May he do good, 287
Leeds General Infirmary, 399
——— Infirmary and Working Men, 435
——— Workpeople and Hospitals at, 28, 222, 251
——— Small Pox at, 284
Leicester, Small Pox at, 183
——— Infirmary, 300
Leicester and Rutland County Asylum, 416
Life's March, A Halt in, 213
Limits of a Nurse's Vocation, 245, 263, 281, 295
Linen and Calico, 49
Liquid Foods, 84
Lind-Goldsmidt, Madame and Worcester Infirmary, 182
Liverpool Royal Infirmary and Earl Derby, 96, 150
——— Emigration of Pauper Children at, 235
——— Dispensaries and Board School Children, 383
——— Bishop of, and Derbyshire General Infirmary, 132
Locum Tenens Question, 11
London, a Teaching University for, 421
London Hospital, New Buildings at, 42
——— Secretaryship, 168, 284, 301
London Fever Hospital, Treatment of Patients, 236
Love Lights, 349
Lunatics, Cruel Treatment of by Parents, 17, 38
Lying-In Hospital, Alleged Red tape at a, 351
——— The City of London, 350
——— Hospitals, 437
Lying-In Hospitals, Origin, History, and Present State of Metropolitan, 398

Makers or Labellers, 421
Material for Underclothing, 117
Matrons' Corner, 12, 87
Medical Assurance Annuity Society, 330
Medical Journalism, 261
Medical School, the, and the Hospital, 356
Medicine, the Oracles of, 1
Medicine, the Dignity of, 293
Medicine Men of the World, 30, 82, 111, 155, 184, 307, 394
Medico-Chirurgical Society of Aberdeen, 335
Men and Marriage, 231
Microbes, 11
Middlesboro' Demonstration of Trade Societies, 8
Midwifery, Free Training in, 438
Midwives, Amelioration of the Condition of, 258
Mission to Deep Sea Fishermen, 253, 269
Modern Drug Laboratories, 129, 162
Montrose Royal Asylum, 148
Monument of the Queen's Reign, the Noblest, 229
Morals of the Charitable, 353
Morals of the Workshop, the, 407
"Morley House" Seaside Home, 285
More Home Claims, 135
Mutual Friendly Aid Society, 98

National Hospital for the Paralysed and Epileptic, 318
National Pension Fund for Hospital Officials, 17, 33, 39, 59, 62, 65, 67, 80, 94, 112, 136, 178, 279, 297, 317, 327, 328, 349, 366, 397, 408
New Wars, New Weapons, 45
Newcastle Hospital and Working Men, 133
Nice Nursing Home, the, 434
Night Attire, 373
Notes for Practitioners, 283
Northampton, Mass. Lunatic Asylum, 365
North-West London Hospital, 148
Not Alms, but a Friend, 173
Norfolk and Norwich Hospital, 209, 234
Norwich City Asylum, 425
Norwich Collection of Calculi, 324, 380
Nursery Cares, 315
Nurses, a Word to, on Registration, 405
Nurses and Sick Soldiers, 37
Nurses, Registration of, 56, 73, 205, 222, 341, 405
——— Hours of Work of, 96, 114
——— Male, for Men, 167
——— Hints for Probationers, 172b
——— Rest for, 200
——— Home Rule for, 227
Nurse's Vocation, the Limits of a, 245, 263, 281, 295
Nurses' Association, the British, 202, 351, 397, 405, 406, 419, 434
Nurses' Certificates, 292, 341
Nurses, Talks with and Aids to, 303, 331, 347, 363, 379, 395, 409
Nurses, Homes Abroad for, 389, 410
Nursing Engagements in the Winter, 389, 410
Nursing, the Art of, 410
Nursing Society, East London, 383, 396
Nursing in the 16th Century, 15, 83
Nursing Sisters, Homes in the Hills for, 163
Nursing the Sick, a Noble Profession, 172b
Nursing Mirror, the, 226, 252, 292, 303, 325, 341, 373, 389, 410, 434, 438

Oberbrunnen, a New Mineral Water, 276
Opportunity, a Great, 169

Paddington, Precept and Example at, 401
Parkes Museum, 148, 150
Patients, a Nurses View of Hospital, 49
"Pathies," the Battle of the, 277
Pauper Children in Liverpool, 235
Pauper Children, Boarding Out of, 221
*Pall Mall Gazette* the, and the Hospitals, 107, 134
Penalties of Weakness, 393
Pedagogues, the World and its, 415
Pension Fund, the National, for Hospital Officials, 17, 33, 39, 59, 62, 65, 67, 80, 94, 112, 136, 178, 279, 297, 317, 327, 328, 349, 366, 397, 408, 444
——— and the Charity Record, 97
Perth, Medical Science at, 53
Perth Infirmary, Small Pox at, 62, 87
Phonograph, the, 151
Photographs for Hospitals, 429
Philosopher, a Medical, 337
Physic Bottle, the, 175
Physicians, the College of, and the *Lancet*, 431
Police, Convalescent Home for Metropolitan, 428
Poor Children's Aid Society, 428
Poor, the, and their Doctors, 312
Portable Hospitals, 334
Portsmouth, Royal Hospital at, 44
Portsmouth Borough Asylum, 416
Port Said, Lady Strangford Hospital at, 221
Practitioner's Clinic, the, 267, 333
——— Notes for, 285
Precocity and Health, 135
Preston Infirmary, Inspection by Working People, 8
Principles of Progress, 359
Printing Trade, the and the Hospitals, 114
Prize Competitions, 33
Professional Notes by Geo. W. Potter, M.D., 25, 41, 77, 113, 147, 179, 219, 249
Probationers and Kitchen Work, 62
——— Health of, 87
Progress in Co-operation, 237
Provident Dispensaries in Birmingham, 412
Provident Dispensaries and the Hospitals, 200, 401

Queen, the, and the National Consumption Hospital, 412
Queen's Hospital, Birmingham, 26, 413
Queen's Jubilee, and the Suffolk General Hospital, 428
Queen's Reign, the Noblest Monument of, 229
Quinn Bequest, the, 413, 419

Rachel Challice's Vow, by Author of "Juno," 255, 273, 289, 305, 323, 339, 354, 370, 387, 402, 417, 432, 447
Recollections of Hospital Life, 177, 241
Red tape, Alleged, at the Lying-In Hospital, 351
Redruth Hospital for Women, 250
Registration of Nurses, 56, 73, 205, 222, 341, 405
Relation of the Medical School to the Hospital, 356
Reviews: Consumption, Climatic Treatment of, 4
——— "Pulmonary Consumption," 146
——— "Katharine Regina," 146
——— "St. Bernard's," 217
——— "Descriptive Anatomy," 308
——— "Elizabeth Gilbert, and Her Work for the Blind," 309
——— "Health for the People," 309
——— "Serbelloni," 309
——— "English Illustrated Magazine," 309
——— "A Prayer Book for Hospitals," 371
——— "The Care of Common Diseases," 371
——— "Surgery for Nurses," 372
——— "Vaccination Vindicated," 372
——— "The Massage Case," 442
——— "Bournemouth and its Surroundings," 443
——— "Organic Materia Medica," 443
Richmond Hospital, 335
Royal Asylum, Montrose, 148
Royal Hospital at Portsmouth, 44
Rollit, Sir A., M.P., and the Hospitals, 229
Royal Medical Benevolent College, 319
Round about the Asylums, 365, 416
Ruabon Cottage Hospital, 202

St. George's Hospital, Legacy of £100,000, 132
——— Cost of Administration at, 134
St John's Hospital for Skin Diseases, 237, 419
St. Mary's Hospital Medical School, Abstract of Introductory Lecture, at, 24
——— Lord R. Churchill and, 412
St. Peter's Hospital, Bristol, Extracts from Record 114
Salisbury Hospital Saturday at, 26
Salt Water Supply for Edinburgh, 151
Scarlet Fever, 55
Scarlet Fever Convalescents, and Mr. Gladstone, 215, 220

Scarlet Fever Epedemic in London, 43, 60, 79, 116
Science and Socialism, 35
Science of Superstition, 151
Science well Paid, 302
Scientific Preacher, a, 337
Schools, Collection Boxes in, 367
Scotch Physicians and Surgeons, 246, 291, 316, 330, 362, 381, 411, 426
Seamen's Hospital, Greenwich, 234, 284, 382
Sheffield, Small Pox Hospital, 27, 132
Sheffield, Small-pox at, 8, 150, 251, 367, 389
Sick, Healing the, 37
Sick Soldiers and their Nurses, 37
Sick, the, the Churches, and the Hospitals, 89, 91, 112, 145
Skin Diseases, St. John's Hospital for, 237, 419
Small-pox Epidemic at Leeds, 284
Small-Pox at Leicester, 183
Smoking on the Episcopal Bench, 43
Sociology, An Experiment in, 19
"Something Attempted," A Retrospect, 244
*Spectator*, the, and Sir J. Paget, 415
Spirit Bottle, the, 197
State Supported Doctors, 183
Street Collections for Hospitals, 80
Students' Column, 100, 218, 332, 348
Surrey Needlework Guild, 181
Suffering Children, 253
Sunday Cot at the Kilburn Hospital, 399
Supper and Sleep, 21
Sweating, 446

Talks with Nurses, 313, 331, 347, 363, 379, 395
Taunton, Mass., Lunatic Asylum, 365
Temperance and Medical Men, 319
*Times*, The, and the Queen's Jubilee, 271
*Truth* Toys for Hospitals, 44

Underclothing, Material for, 117
Unemployed in London, the Problem Solved, 159, 166a, 173, 193
University College Hospital, 148, 268

Vaccination, Is it a Failure? 46
Vegetarianism, 446
Victoria Jubilee Hospital, Bournemouth, 383
Victoria Hospital for Children, 222
——— Advertisements in Annual Report of, 134
Village Life, Miss Twining on, 29
Virtuous Journalism, 282
"Voluntaries," by D. Stott, 42

Wakefield, Clayton Hospital, Annual Open-air Sunday Concert, 9
Warwick Asylum, Charges Against, 98
"Way of Life," Professor Huxley's, 376
Well-paid Science, 302
Westminster Hospital and the Jubilee, 318
What dreams are made of, 353
Whitaker's Almanac and the Hospital, 98
Wicked or Weak, 369
Woman at Home, 91, 109, 127
Woman at Home, a woman on, 128,
Women, a new occupation for, 353
——— Chelsea Hospital for, 334
——— Redruth Hospital for, 250
Women's Jubilee Offering, the, 271
Words of Consolation :
——— The Sufferings of Christ, 2, 20, 36, 54, 72, 90, 108, 126, 142, 174, 244, 278, 294, 312, 344, 360, 376, 392, 422, 438
——— Peace and Goodwill, 274
——— The End and the Beginning, 228
Worcester Infirmary, and Madame Lind-Goldsmidt, 182
Worcester Infirmary, and the Bishop of the diocese, 219
Working-class view of our Hospitals, 257, 264, 288, 296, 314
Work People's Subscriptions, 28, 76, 222, 251
Workington Infirmary and Working-men, 350
Working-men, and the Newcastle Royal Infirmary, 429
Working-men Governors at Hull Infirmary, 150
Workmen's Contributions to Leeds Infirmary, 435
Work for the Unemployed at Paddington, 401
Work, the Medicine of, 329

### NOTICE TO ADVERTISERS.

THE scale of charges for small prepaid Advertisements—Situations Wanted, Situations Vacant, etc., etc., is as follows :—

| | s. | d. |
|---|---|---|
| One insertion of three lines | 1 | 0 |
| Three „ „ „ | 2 | 6 |
| Per line afterwards | 0 | 6 |

*A line averages ten words.*

All copy for Advertisements must be sent to A. P. WATT, 2, PATERNOSTER SQUARE, E.C., by first post on the Tuesday *preceding* publication.

### NOTICE TO CORRESPONDENTS.

All correspondence must be written on one side of the paper only.
We cannot undertake to return MSS. not used.

**Communications,** whether intended for insertion or for private information, must be authenticated by the names and addresses of the writers, not necessarily for publication.

**Local papers** containing reports or news paragraphs **should be marked.**

It is especially requested that early intelligence of **local events of interest,** or which it may be thought desirable to bring under notice, may be sent direct to the Editor.

Communications respecting editorial matters should be addressed to the Editor, Norfolk House, Norfolk Street, Strand, London, W.C.

### AMUSEMENTS WITH PRIZES, FOR ALL AGES.

### SPECIAL NOTICE.

DURING the past year we have offered upwards of 30 guineas in prizes, of which upwards of twelve have been for children, who have come forward in considerable numbers. For some reason the competitors for the remaining twenty pounds have been comparatively so few as to make it doubtful whether to continue these prizes beyond the 15th inst., when a new series is due to commence. We are disposed to continue the experiment for another year, if there is any general wish amongst the readers of THE HOSPITAL that this shall be done. Few at present appear to realise that it is possible to earn three guineas a quarter and five guineas once a year under the present scheme. We shall therefore give an extra prize of one guinea to the correspondent who sends the names and addresses of the greatest number of subscribers, old or new, who will undertake to enter for the prizes offered to men and women during the ensuing year, and a further prize of one guinea for the best suggestion for increasing the interest in these competitions. These prizes are subject to the condition that at least twenty competitors enter for each of them, and that the lists and suggestions reach us not later than Monday, the 17th inst. All answers should be addressed to the Editor, THE HOSPITAL, Norfolk House, Norfolk Street, London, W.C., and have distinctly written in the left-hand corner of the envelope the words "New Prize Competition." The *nom de plume* of all competitors for these special prizes will be published on 22nd October with the results, and our decision for the coming year.

### FOR MEN AND WOMEN.

**Quarterly Prize Competition.**
Began 23rd July; ends 15th October.

**A Prize of Three Guineas**
will be awarded to the Competitor who shall have sent in the largest number of correct or best answers.

**A Prize of Five Guineas**
to the competitor who shall have sent in the largest number of correct or best answers during the twelve months.

**No. 21. Double Acrostic.**

It dropt beside my first, and rolled upon the ground,
My finals on him burst, the law he sought was found.

He wrapt his mantle round him, and alone
Passed forth unrecognised, because unknown.

Our wicked works thou cuttest short,
Light from their dimness to extort.

Haggard and wan, you ask,
"What can it be
Confronts me grand and ghostlike?"
'Tis, 'tis She!

I'm at sea, quite at sea, to find my fourth;
And the sea is black, and the light lies north;
So I'll steer thro' the Strait, as straight may be,
And find my fourth light, tho' I'm still at sea.

A gentle, meek reformer
Touch'd the blot
But touching, fell disgraced,
Rome changeth not.

What can you make of this light?
Nothing. 'Tis vacancy quite.

Flashes of wit! sparkles of fun!
Satire and skit! pathos and pun!
Jew, gentleman, angel, compounded in one.

What with shuffling, sharp dealing, and tricks you'd suspect
With knaves only I reckon'd should be;
But kings, ay, and queens, my condition affect,
And always bring honours to me.

Dies sweet cadence of the solo string:—
The maestro's dark eyes flash—
His swift white hands the message fling,
And hark! the orchestral crash.

Creation of a dream,
Great Manfred's castle rose,
Where spectral hands administer'd
Hereditary woes.

Sans smell, sans taste. 'Tis everywhere
Impalpable, prime element of atmospheric air.

**No. 22. Mathematical Problem.**

A certain number is divided into four parts. To the first part you add 2, from the second part you subtract 2, the third part you multiply by 2, and the fourth part you divide by 2: and the *sum* of the addition, the *remainder* of the subtraction, the *product* of the multiplication, and the *quotient* of the division are all equal and precisely the same. How is this?

**Answers.**

No. 17. DOUBLE ACROSTIC.

S enn A

P er U

R es T

I mprompt U

N iflhei M

G riffi N

Correct answers have been received from Condorcet, Mrs. Bedingfeld, Mater, Gretta, Brownie, Esau.

No. 18 ENIGMA.

The answer to this is *Snuffers*, which Esau alone has solved correctly.

### THE CHILDREN'S CORNER.

**First Monthly Prize Competition.—Second Year.**

Begins 1st Oct., 1887. ends 29th Oct., 1887.

PRIZES.
FOR MOST CORRECT ANSWERS TO PUZZLES.
One Pound per Month.
FOR BEST LITTLE LETTERS.
Ten Shillings per Quarter.
A PRIZE OF THREE GUINEAS
to the Competitor who shall have sent in the largest number of correct or best answers during the twelve months.

**Puzzles—First Week.**

No. 1. HISTORICAL CHARADE. My first and second are an adjective signifying sacred; my third a measure of land; my whole an ancient and historical castle in Scotland.

No. 2. FLORAL ANAGRAMS. 1. One name. 2. Set claim. 3. A sore chin. 4. Scan a hut.

No. 3. RIDDLE-ME-REE.

My first is in house, but not in hut;
My second is in pear, but not in nut;
My third is in day, but not in night;
My fourth is in wrong, but not in right;
My fifth is in age, but not in youth;
My sixth is in error, but not in truth.

No. 4. CONUNDRUM. What poet offers a refuge to lions?

**Letters—First Week.**

Next week, the letter-subject will be your favourite "Hero" or "Heroine" in history.

**Results of Twelfth Week—Puzzles.**

No. 46. HISTORICAL ACROSTIC.

P oictiers
A gincourt
V ittoria
I vry
A usterlitz

No. 47. MISSING VOWEL PUZZLE.

"Cans't hear," said one, "the breaker roar,
For methinks we should be near the shore;
Now where we are we cannot tell,
But I wish I could hear the Inchcape bell."

No. 48. BURIED ISLANDS. 1. Borneo. 2. Andaman. 3. Cyprus. 4. Lewis.

No. 49. The French towns denoting an ill-fitting garment were easily discovered by all my young friends; they are Toulon (Too long), Toulouse (Too loose).

*All right*—A. C. K., Poodle, Agatha, Umslopogaas; *Three right*—Mount Edgcumbe, Skye, Ossian, Scrooge, The Eda, Toby, A. G. S. McCulloch; *Two right*—Princess Ida, Fern.

**Letters.**

I have received replies on various topics in response to my request that each boy and girl should choose their own subject for this week's letters. Mount Edgcumbe describes the delights of life in Tasmania, and gives some interesting details of the history of the colony, and the animals and plants found there. Socrates writes on horses; Toby, of the treasures in the Kensington Museum; Princess Ida, on her favourite authors; Ossian, on letter-writing in general, and the penny postage in particular; whilst Skye chooses a "country walk in autumn," and The Eda the subject of picnics, which we have had before.

Three marks each:—The Eda, Toby, Socrates, Princess Ida, Skye, Ossian, Mount Edgcumbe.

**Rules.**

All answers must be addressed to THE PRIZE EDITOR, THE HOSPITAL, Norfolk House, Norfolk Street, Strand, W.C., not later than the *first* post on Friday next. The full name and address must in all cases be given but a *nom de plume* may be added for publication. Only one side of the paper should be written on.

No one will be permitted to win the Monthly or Quarterly Prizes twice in any one year, the annual prizes being offered especially to prevent this.

